# Quantification of Myocardial Deformation Applying CMR-Feature-Tracking—All About the Left Ventricle?

**DOI:** 10.1007/s11897-021-00515-0

**Published:** 2021-05-01

**Authors:** Torben Lange, Andreas Schuster

**Affiliations:** Department of Cardiology and Pneumology, Georg-August University Göttingen and German Centre for Cardiovascular Research (DZHK), partner site Göttingen, University Medical Center Göttingen, Robert-Koch-Straße 40, 37075 Göttingen, Germany

**Keywords:** Cardiovascular MRI, CMR-feature-tracking, Myocardial deformation analysis, Myocardial strain, Improved risk stratification

## Abstract

**Purpose of Review:**

Cardiac magnetic resonance-feature-tracking (CMR-FT)-based deformation analyses are key tools of cardiovascular imaging and applications in heart failure (HF) diagnostics are expanding. In this review, we outline the current range of application with diagnostic and prognostic implications and provide perspectives on future trends of this technique.

**Recent Findings:**

By applying CMR-FT in different cardiovascular diseases, increasing evidence proves CMR-FT-derived parameters as powerful diagnostic and prognostic imaging biomarkers within the HF continuum partly outperforming traditional clinical values like left ventricular ejection fraction. Importantly, HF diagnostics and deformation analyses by CMR-FT are feasible far beyond sole left ventricular performance evaluation underlining the holistic nature and accuracy of this imaging approach.

**Summary:**

As an established and continuously evolving technique with strong prognostic implications, CMR-FT deformation analyses enable comprehensive cardiac performance quantification of all cardiac chambers.

## Introduction

**Fig. 1:**
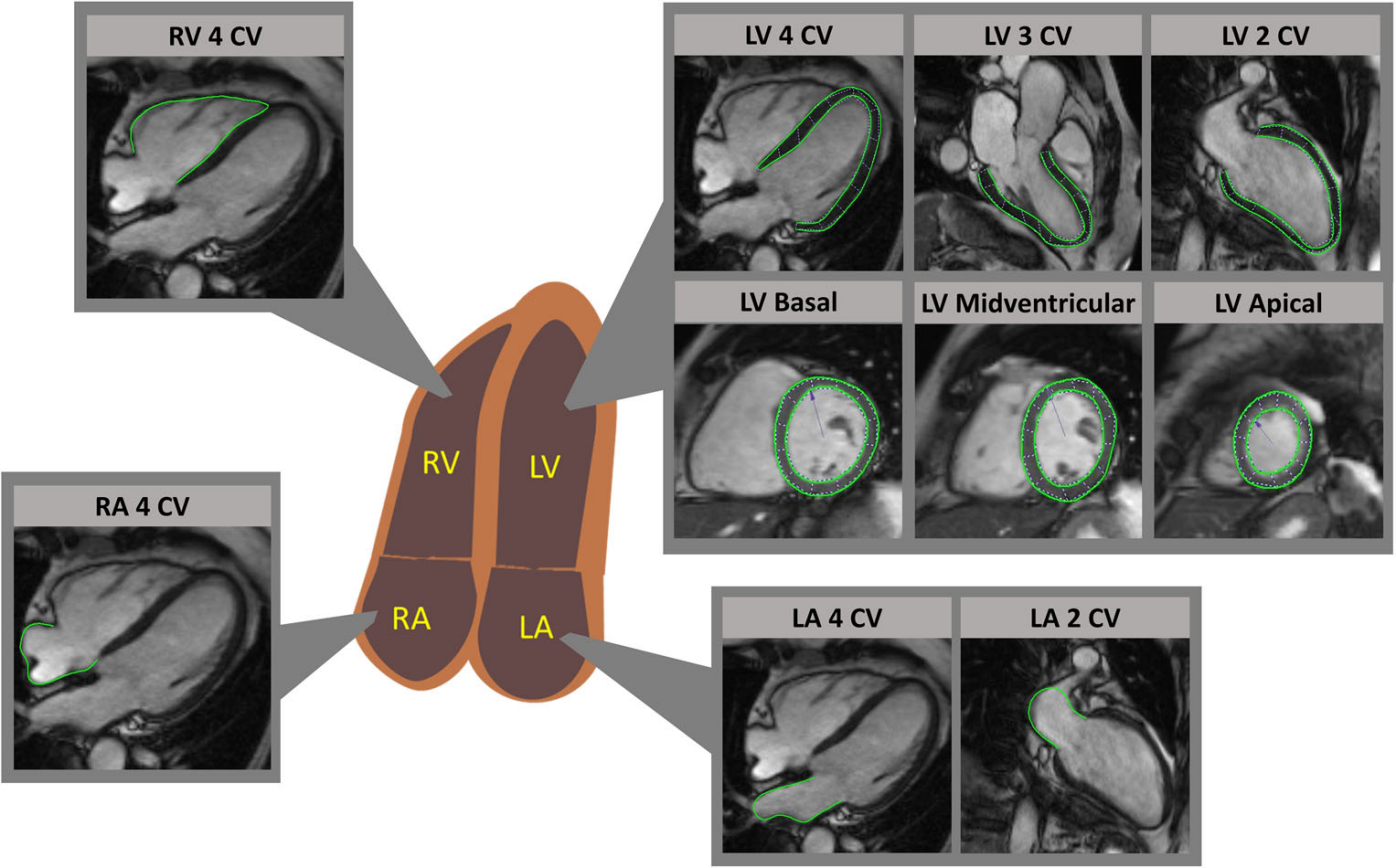
Cardiac magnetic resonance-feature-tracking across all cardiac chambers across all cardiac chambers. Exemplary feature-tracking of left and right ventricles (LV & RV) in long-axis orientations of 2-, 3- and 4- chamber views (CV) and LV contours in a short axis stack from base to apex. Left and right atrial (LA & RA) delineations are displayed in long-axis based 4- and 2-CV images

Cardiac imaging plays a key role in the diagnostic algorithm of heart failure (HF), which is one of the major challenges in cardiovascular medicine [1, 2]. As a complex clinical syndrome comprising a wide range of symptoms and consequences (dyspnea, congestion, organ failure), the impact of HF on patients’ prognosis and outcome is substantial. To date, left ventricular (LV) performance and left ventricular ejection fraction (LVEF) are most commonly quantified to assess cardiac function in cardiovascular diseases. However, with several potential underlying causes and multifarious pathophysiological mechanisms for HF, precise diagnostics are complex and preserved LVEF does not automatically reflect preserved myocardial function [3]. Furthermore, both myocardial structural and functional abnormalities are decisive for systolic and diastolic dysfunction affecting not only LV integrity but also right ventricular (RV) or atrial performance that can be affected by the respective cardiac disease itself or influenced by LV failure with secondary congestion and volumetric alterations. Thus, deformation analyses beyond the LV are moving into the spotlight and modern cardiac imaging of heterogenous forms of HF is required to capture and depict all HF facets as comprehensively as possible for a precise evaluation of functional impairment processes and to assure optimal patient management. In this context, cardiac magnetic resonance (CMR) imaging has evolved into a well-regarded, established and beneficial imaging technique over the last years. The unique ability for extensive analyses of both cardiac deformation, volumes, myocardial morphology and tissue composition with growing evidence of its prognostic importance and superiority over currently used clinical parameters makes CMR imaging an excellent and powerful tool for appropriate HF quantification [4]. Especially CMR-derived analyses of LV deformation and beyond have been subject of numerous recent studies and are of increasing importance in the scope of clinical imaging (Fig. 1). In the present review, we consequently outline the current role of CMR-based deformation analyses in cardiovascular diagnostics of HF, its fields of application over and above LV analyses, clinical benefits and provide perspectives for future developments and utility.

## Techniques, Basic Principles and Measurement Capabilities

**Fig. 2:**
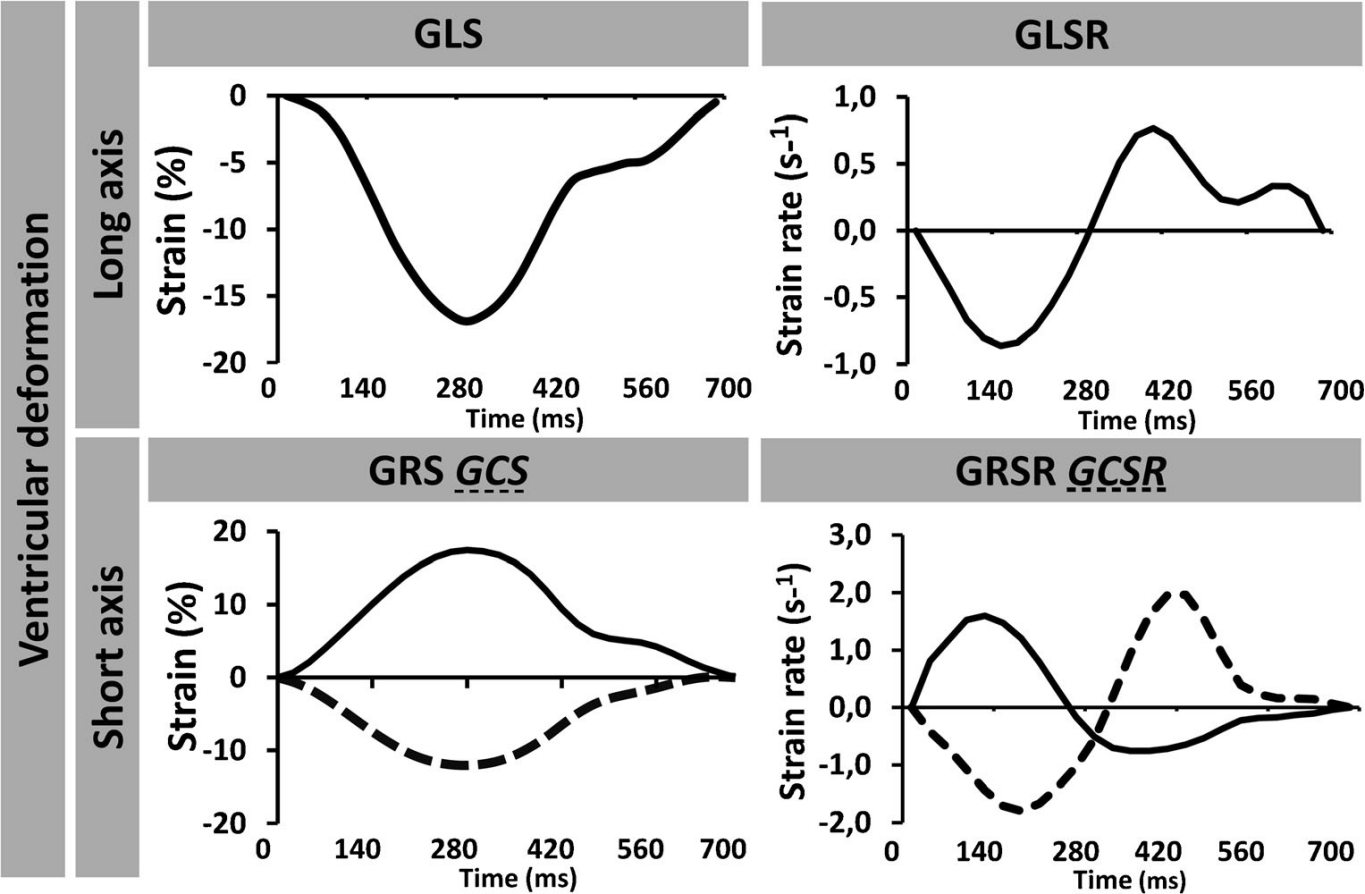
Cardiac magnetic resonance-feature-tracking-derived ventricular strain and derived ventricular strain and strain rate. Exemplary curves of ventricular long-axis views generating global longitudinal strain (GLS) and corresponding strain rate (GLSR) values as well as quantification of global circumferential strain GCS (dotted line) and GRS (solid line) with corresponding strain rates (GCSR and GRSR)

Since first reports from CMR-based deformation analyses in 1988 [5], techniques have undergone a considerable development and CMR parameters have become established imaging biomarkers in clinical practice [6]. Currently, different approaches for CMR-based myocardial deformation analyses are available. Myocardial tagging or strain-encoded (SENC) imaging was amongst first attempts of analysing myocardial deformation; however, due to long acquisition and post-processing times or limited availability of required sequences, clinical implementation is hampered yet despite several technical refinements and improvement efforts [7]. In contrast, CMR-feature-tracking (CMR-FT) is based on routinely acquired ECG triggered SSFP CMR images that deliver high contrasts between blood pool and myocardial structures [8], enabling simple clinical implementation and extensively validated strain and strain rate (SR) deformation assessments [9–11]. Recent developments in CMR techniques even enable real time (RT) imaging with high temporal resolution, similar image quality and spatial resolution compared to conventional SSFP images [12, 13]. For the procedure of CMR-FT, after precise delineation of epi- and/or endocardial borders, a semi-automated algorithm of the FT software tracks the myocardial border displacement over the whole cardiac cycle by utilizing an optical flow technology that measures motion vectors of predetermined points [14]. As a result, spatial motion profiles of myocardial interfaces can be generated and displayed along a time axis. Additional complex processing of data including spatial coherence, signal/noise ratio, frame rate or temporal and spatial resolution subsequently enables calculation of comprehensive deformation analyses [15]. In this way, longitudinal strain can be calculated in long-axis views, whereas circumferential and radial strain values can be derived from LV short-axis orientations (Fig. 2).

**Fig. 3:**
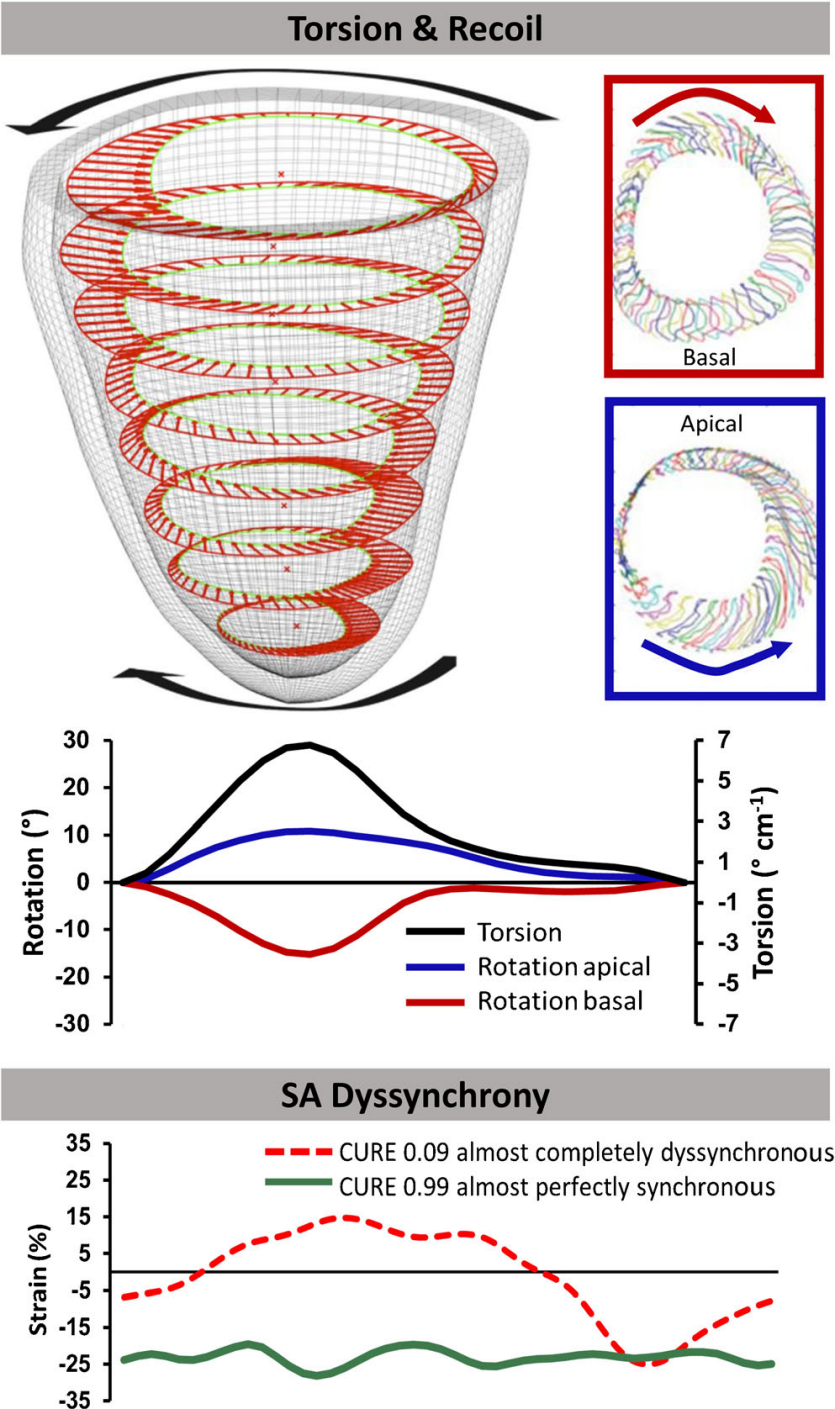
Cardiac magnetic resonance-feature-tracking-based rotational rotational mechanics and measurement of strain dyssynchrony. Shot axis (SA) based myocardial torsion with clockwise basal (red) and counter-clockwise apical (blue) rotation (when viewed from the apex) divided by the distance between both slices generating the myocardial torsion. Oscillations of circumferential strain plotted against spatial positions expressing myocardial dyssynchrony. Values of exemplary circumferential uniformity ratio estimate (CURE) range between 0 representing complete dyssynchrony and 1 perfect synchrony. Parts of this figure are adapted and originally taken from Kowallick et al. (15) and provided under the terms of the Creative Commons Attribution 4.0 International License (http://creativecommons.org/licences/by/4.0/)

Besides LV strain evaluations, CMR-FT also has been shown to enable measurement of LV tissue velocity, rotational mechanics (calculating angular movements of a tracked point between systole and diastole with regard to the centre of gravity) or circumferential and radial uniformity ratio estimates quantifying synchrony of myocardial deformation by plotting strain against spatial position and measuring oscillations as an expression of myocardial dyssynchrony [16–19] (Fig. 3). These analyses considerably expand the possibilities of displaying LV mechanics adding important information of deformation, that is not captured by sole strain assessment.

**Fig. 4:**
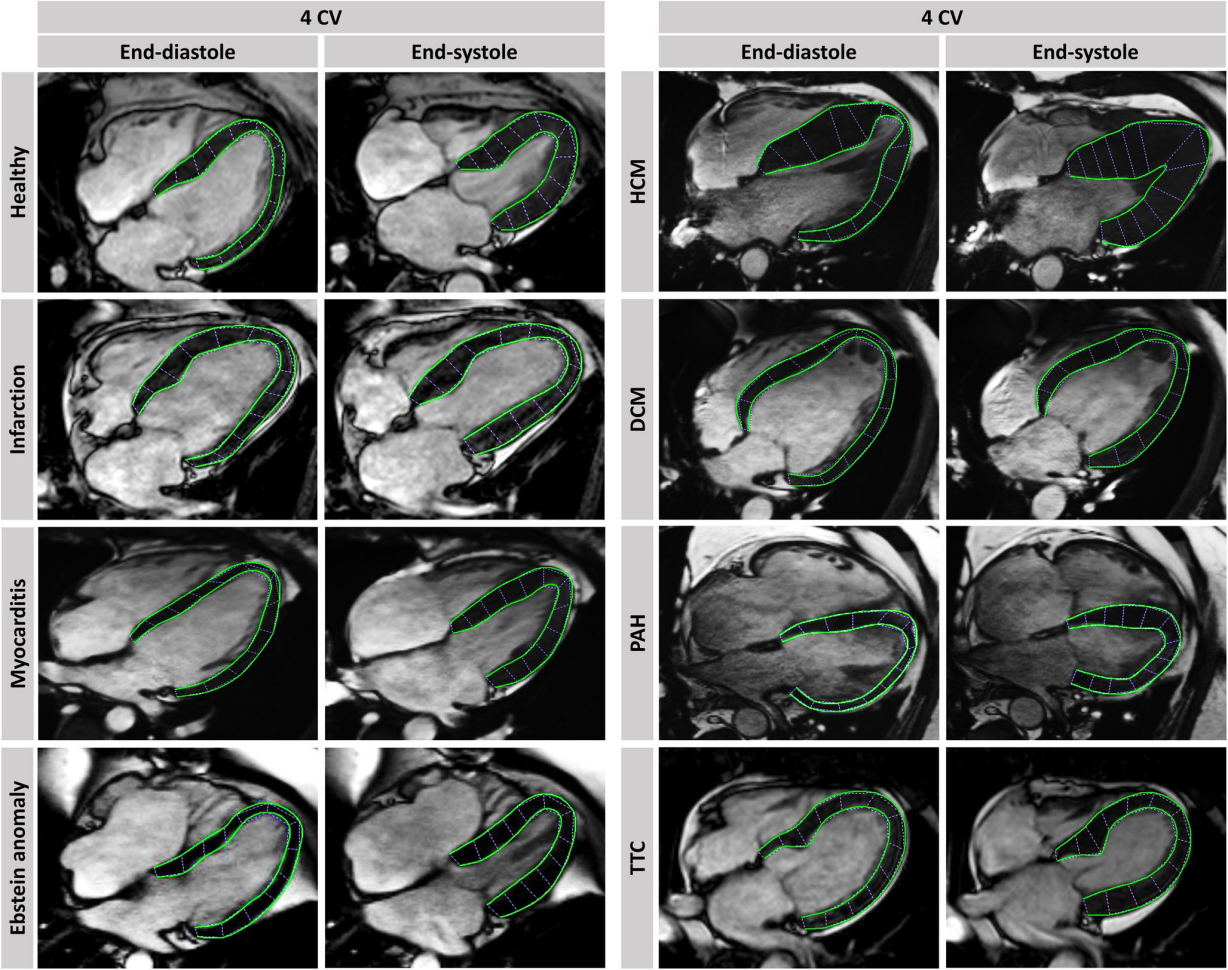
Cardiac magnetic resonance-feature-tracking in different diseases. Exemplary LV epi- and endocardial borders in 4 chamber view (CV) long-axis orientations are tracked in end-diastole and –systole. HCM: hypertrophic cardiomyopathy; DCM: dilated cardiomyopathy; PAH: pulmonary arterial hypertension; TTC: Takotsubo cardiomyopathy

By now, many studies have shown excellent feasibility and reproducibility of CMR-FT analyses tools [20–22] defined normal value ranges [23, 24], investigated inter-vendor agreement of different available software vendors [25–27] and examined it in a wide range of cardiac diseases demonstrating their applicability and clinical utility (Fig. 4). It is important to note that besides excellent intra- and interobserver reproducibility for FT-derived global strain assessments, interchangeability between different vendors is limited and needs to be considered when comparing FT-parameters derived by different software packages [20]. Harmonisation of software algorithms or specific correction factors might address this issue for a better comparability in the future [28].

## Deformation Quantification Beyond LV

**Fig. 5:**
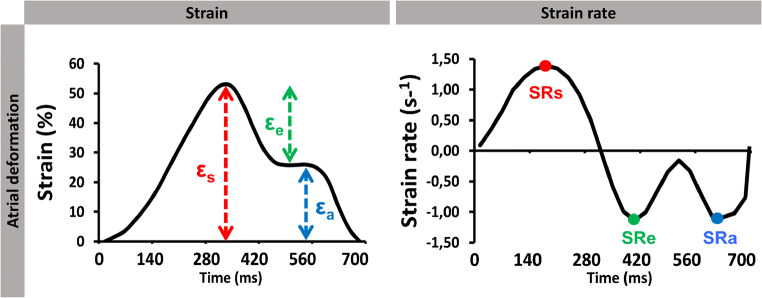
Cardiac magnetic resonance-feature-tracking-derived atrail strain and strain rate (SR). Atrial functional strain and SR measures comprising reservoir (εs & SRs), conduit (εe & SRe) and booster pump (εa & SRa) function

Even though most CMR-FT studies predominantly focused on LV deformation analyses for HF quantification, there is growing literature and evidence of extending CMR-FT analyses beyond LV performance assessment. Quantification of RV and atrial deformation is challenging due to thin-walled anatomy, complex geometry and single plane analyses especially of the right heart, but CMR imaging has been shown to offer great potential for accurate assessment of these cardiac compartments [15]. Consequently, both RV [29, 30] and atrial [28, 31, 32] deformation analyses are coming to the fore in current CMR-FT-based research, proving feasibility, generating reference values, demonstrating reproducibility and utilizing RV as well as atrial CMR-FT parameters in various cardiac diseases. In detail, similar to LV GLS analyses RV longitudinal strain evaluations can be derived from 4-chamber long-axis views. Furthermore, atrial performance assessments are feasible by generating strain and SR parameters of three atrial functional components: (1) reservoir function representing the collection of pulmonary venous return during ventricular systole, (2) conduit function during passive passage of blood to the left ventricle at early diastole and (3) booster pump function as the augmentation of ventricular filling during late diastole by active atrial contraction [31] (Fig. 5). Although there is an inevitable association between atrial and ventricular physiology [33, 34], atrial myopathy has been introduced as a novel term and an own entity dissociating the integrity of atrial performance from ventricular function and compliance [35]. According to these concepts, atrial dysfunction can be considered not only as a surrogate for LV failure caused congestion and alterations in atrial volumes but also as a manifestation of atrial deficiency itself for maintaining normal contractile function or unrestricted filling [36]. Consequently, independent deformation quantification beyond LV assessment can contribute to a more complete quantification of myocardial function and a more in-depth understanding of pathophysiological processes in HF. On the growing basis of its practicability, applications across various myocardial diseases are rising, providing new insights with diagnostic and prognostic implications.

## Deformation Analyses in Acute and Chronic Ischemia

Cardiomyopathies are common causes of HF. Timely diagnosis, continuous evaluation of myocardial function and accurate risk stratification are essential to identify appropriate therapeutic strategies and to reduce cardiac mortality. As one of the most important causes for HF, acute myocardial infarction (AMI), coronary artery disease and ischemic cardiomyopathy (ICM) have been in the focus of several CMR-based deformation studies [37]. CMR-FT-based LV global strain values and especially global longitudinal strain (GLS) have been identified as powerful and independent predictors of adverse events and mortality in patients with AMI and ICM [38, 39]. In addition, as a rapid and widely available approach for LV performance, fast long-axis strain (LAS) has been applied in AMI patients and revealed an easy and fast approximation with incremental prognostic value in these patients [40]. In this way, a simplified deformation analysis with high reproducibility is possible [41] and analyses even without dedicated post processing software are conceivable. Importantly, by predicting ventricular arrhythmia or early myocardial remodelling very accurately [42, 43], CMR-FT-based LV strain was found to outperform traditionally used LVEF in these studies further questioning current approaches using a LVEF cut of 35% for defibrillator device therapy and raising considerations of new risk classifications beyond this parameter. Of note, not only global LV strain analyses but also regional CMR-derived strain evaluations of non-infarcted remote myocardium were shown to allow an extended risk classification and categorise new high-risk groups in patients suffering AMI [44].

Besides LV strain assessments, alterations of CMR-FT-derived myocardial LV uniformity as novel markers with important additional prognostic implications have been introduced [45]. Especially in patients with a LVEF > 35%, uniformity ratio estimate values enabled an independent risk prediction underlining the important aspect of ventricular performance impairment in the form of mechanical dyssynchrony that is not sufficiently reflected neither by LVEF nor LV strain. In this way, AMI caused subtle subendocardial fibre damages can be more sensitively detected by uniformity analyses and widen the potential of CMR-FT assessments to improve post-infarction risk stratification. Although major benefits of predominantly LV CMR-FT deformation analyses over established clinical parameters have been reported in numerous studies, the importance of right ventricular (RV) and atrial function for precise diagnosis and improved risk stratification is increasingly recognized. Depending on the culprit vessel, specific characterisation of morphologic and functional impairments after AMI can be detected across cardiac chambers by CMR-FT evaluations [46]. The RV is largely supplied by the right coronary artery and an occlusion can affect its integrity in almost 50% of these patients [47], which is why an evaluation of the RV performance is indispensable in both acute and chronic myocardial ischemia. Indeed, an impaired RV deformation has been demonstrated in patients with AMI using CMR-FT and RV GLS was found to be an important additional predictor of adverse events over and above RV tissue characterisation, consequently considering this CMR-FT parameter for an optimised post-infarct risk management as well [48].

Directly adjacent to the LV, LA performance is inevitably influenced by LV function but has the potential to compensate initial ventricular failure after AMI by an increased booster pump function. However, subsequent stress-induced LA contractile failure can lead to cardiopulmonary congestion and distinct HF symptoms [49]. Importantly, impaired LA performance was found to be a distinct feature and independent risk factor not only enabling improved diagnostic accuracy but optimizing risk classification in these patients. In particular, LA reservoir strain emerged as the most robust atrial functional parameter and has been shown to have significant associations with adverse outcome and to enable the identification of additional groups of patients at high risk after AMI [49, 50].

Furthermore, there is evidence of the utility of right atrial (RA) deformation analysis to completely assess the involvement of the right heart after AMI. Similar to LA deformation evaluations, RA strain analyses showed significant associations with adverse events after AMI and provided additional risk stratification [51]. Not only does impaired RA performance hamper diastolic filling of the RV and subsequently LV preload, but it is also directly related to pulmonary artery pressure, which is, in turn, influenced and potentially increased due to LV failure. Therefore, RA dysfunction can either represent ischemic right heart involvement and/or reflect consequences of LV congestion and therefore is another important element of comprehensive HF assessment. Furthermore, despite atrio-ventricular coupling and dependence on ventricular capacity, the proven independent association of atrial performance with adverse outcome underlines the considerations of a unique atrial cardiomyopathy with autonomous resistance and capabilities for compensation and accentuating the crucial role of atrial deformation analyses in ischemic HF.

Importantly, CMR technique offers unique capabilities beyond deformation analyses in patients suffering ICM by enabling profound myocardial tissue analyses. These myocardial characterisations include visualization of edema, infarct, microvascular obstruction, late gadolinium enhancement (LGE) for scar quantification well as myocardial ischemia and viability testing based on dobutamine administration [52, 53]. In addition, CMR imaging can be used for the detection of several complications of AMI like spotting septal rupture, differentiation between an aneurysm or pseudo-aneurysm and identification of thrombi [54] highlighting the broad applicability of CMR imaging and CMR-derived deformation quantification for a wide, multiparametric cardiac assessment and for improved risk stratification in these patients.

## CMR-FT in Non-ischemic Cardiomyopathies

Amongst patients with non-ischemic HF, dilated cardiomyopathy (DCM) is one of the most common phenotypes [55]. CMR imaging was extensively shown to allow precise and accurate diagnosis of this cardiac disease [56]. Similar to findings in ICM, there is substantial evidence of CMR strain analyses as independent and powerful predictors of adverse outcome and mortality in patients suffering DCM [39, 57, 58]. Likewise, fast LAS offers incremental information for the prediction of adverse cardiac events in these patients [59]. Recent approaches including strain assessment even enabled distinguishing between DCM patients and physiological exercise-induced cardiac remodeling by applying CMR stress protocols [60]. Furthermore, CMR strain assessment by measuring low LV contractile reverses under dobutamine stress enabled prediction of biventricular fibrosis [61] and myocardial remodelling in patients with DCM, which was associated with a lower probability of functional recovery [62]. Importantly, biventricular dysfunction and a significant prognostic impact of RV GLS in patients with advanced staged DCM have been demonstrated beyond LV performance quantification [63].

Other cardiomyopathies, in which CMR imaging can significantly contribute to correct diagnosis finding by precisely assessing typical cardiac pathomorphology and FT-derived quantification over and above LV evaluation, were shown to be feasible and provide additional important prognostic information comprising hypertrophic cardiomyopathy (HCM) [64], Takotsubo cardiomyopathy (TTC) [65, 66], restricted cardiomyopathies like amyloidosis and sarcoidosis [67, 68] or arrhythmogenic right ventricular cardiomyopathy (ARVC) [69].

RV strain assessment was shown to be feasible and biventricular involvement indicated by an impaired RV strain was associated with adverse outcome in HCM patients [70, 71]. With HCM not only affecting ventricular morphology but also inducing atrial enlargement, especially LA function and abnormalities have been shown to characterise different stages of the disease. An increase of LV fibrosis but not hypertrophy itself was shown to be paralleled by deterioration of LA reservoir function, whereas LA booster pump was only impaired at later stages of the disease. LA conduit function was already impaired at early stages, therefore, representing the most sensitive LA strain parameter for early detection of fibrosis in HCM patients [72]. As another approach underlining benefits of CMR technique for diagnostic purposes, a multiparametric approach combining various CMR-FT and structural analyses provided important information for discrimination between HCM and athlete’s cardiomyopathy [73]. Interestingly, in exercise-induced hypertrophied athlete’s hearts, both LV and RV strain assessments even revealed differences between athletes of different sport disciplines [74], underlining the importance of comprehensive deformation analyses to capture and classify different exercise-induced myocardial adaptions of all cardiac chambers and to enable a distinction from hypertrophy of life-threatening cardiomyopathies [75].

Due to physical or psychological stress triggers, patients with TTC can exhibit transient contraction abnormalities resulting in different ballooning types, especially of the LV. Besides LV strain as potential parameter for risk prediction [65], CMR-FT-based analyses of dyssynchrony and rotational mechanics revealed increased dyssynchrony that affected rotational movements of the myocardium during the acute phase and were particularly pronounced in patients with apical ballooning pattern. In addition to an improved pathophysiological understanding, these findings were associated with more severe stages of the disease and therefore might possess additional prognostic implications [76]. Furthermore, in a substantial number of TTC patients, RV involvement was observed and associated with adverse outcome [77, 78]. Importantly, CMR-FT strain analyses can identify an impaired RV function and were able to detect RV involvement more sensitively than sole visual evaluation and subsequently enabling an optimised risk stratification in this way [79]. Furthermore, CMR-FT-based ventricular functional impairment has been shown to be accompanied by a transient decrease of atrial reservoir and conduit function reflecting diastolic dysfunction caused by LV ballooning, whereas booster pump strain values were increased in the acute phase of TTC as an expression of an atrial compensation mechanism [80]. Of note, an impaired atrial booster pump was significantly associated with mortality emphasising the important role of atrial function and its ability for compensation of this transient disease and its independent functional and prognostic character.

Focusing on RV performance assessment in ARCV patients, another study not only detected subtle RV strain and SR impairments that were most pronounced in basal levels leading to the assumption that structural changes in ARVC are found to be predominantly located in the subtricuspid region but also proved the feasibility of CMR-FT-derived strain measures to allow the differentiation between ARVC, right ventricular outflow-tract tachycardia as well as Brugada syndrome and healthy volunteers [81].

Thus, by enabling accurate quantification of biventricular and atrial function, providing insights into myocardial morphology, contributing to exact diagnosis and yielding incremental prognostic information, the fundamental role of CMR imaging and FT deformation analyses become apparent across different cardiomyopathies for establishing their etiologies, defining stages of the disease and determining prognostic implications [82].

## Myocardial Inflammation and Other Cardiac Disorders

Acute myocarditis is a challenging cardiac disease with variability in clinical presentation and evolution and therefore complicates timely and exact diagnosis [83]. CMR-FT-based GLS has independent and incremental prognostic value over clinical features like LVEF or LGE and may serve as novel marker for improved risk stratification [84]. Importantly, CMR-based deformation analyses of atrial function were shown to have discriminative power predicting the presence of a myocarditis and exhibited impaired atrial strain performance as an indicator of ventricular diastolic dysfunction [85]. Furthermore, especially combinations of atrial and ventricular strain parameters in addition to established Lake-Louis criteria for the diagnosis of myocarditis improved the diagnostic performance and identified atrial function to be more sensitive detecting early functional changes than sole LV function in this disease. In addition to CMR-FT quantification, multiparametric approaches merging FT-derived strain and CMR-based tissue characteristics like T2-mapping or LGE analyses even widen technical possibilities and diagnostic accuracy in suspected acute myocarditis compared to each imaging parameter alone and demonstrate the importance and special capabilities of CMR imaging technique for comprehensive myocardial analyses [86].

In other relatively rare diseases like Marfan syndrome, Fabry disease or systemic sclerosis that can potentially affect myocardial structures, the use of CMR-FT including biventricular strain assessments and myocardial dyssynchrony was also demonstrated to sensitively detect myocardial dysfunction [87–89]. For example, subtle attenuations of LV and RV longitudinal function were found in patients with Marfan syndrome, while RV GCS values were increased reflecting a potential compensation mechanism and indicating the existence of a Marfan-related cardiomyopathy that is detectable by applying CMR-FT deformation analyses [90]. Furthermore, CMR-FT detected lower RV strain in patients with convalescent Kawasaki disease and RV functional impairment was more pronounced in those with persisting coronary artery lesion [91]. Another study showed high iron deposition in the myocardium to be detectable including CMR-FT-derived RV deformation and therefore timely modification and chelation therapy may be navigated by this non-invasive imaging approach [92]. Subclinical myocardial involvement in the form of both strain impairment and increased dyssynchrony was also detected by CMR-FT analyses in patients with cocaine addiction [93]. Noteworthy, that CMR strain analyses not only enable detection of functional deterioration and adverse influence of substances but also allows monitoring of HF medication like levosimendan and its effects on cardiac function as a commonly administered drug in higher stages of HF [94]. Consequently, in a variety of diseases, CMR-FT-based deformation imaging biomarkers over and above LV assessment allow early detection of myocardial affection and impaired cardiac function before commonly used clinical parameters do and enable accurate process monitoring of HF in this way.

## Diastolic Dysfunction

Amongst HF entities, HF with preserved ejection fraction (HFpEF) plays a particular role and is increasingly recognized by cardiovascular imaging specialists [95]. Diastolic dysfunction is conventionally evaluated by echocardiography; however, the importance of CMR-based comprehensive deformation analyses over and above LV function with the ability of CMR imaging for additional superior assessment of myocardial tissue composition, volumes and masses becomes evident in this clinical picture. Although the full role of CMR is still evolving, it already enables accurate assessment of HFpEF and several studies have applied this technique for a better understanding of HFpEF pathophysiology [96].

On the one hand, comprehensive evaluations of ventricular mechanics have proven CMR-based analyses to detect early diastolic dysfunction, which is not exclusive to HFpEF patients but can also occur in other cardiac diseases and precede LV systolic dysfunction [97]. For example, ventricular SR analyses for the assessment of rotational twisting and untwisting of LV contraction can detect diastolic dysfunction by altered untwisting rates during early diastole [98]. Furthermore, impaired GLS was shown to predict abnormal LV relaxation, to correlate with invasively measured diastolic functional indices and to enable accurate evaluation of diastolic dysfunction in combination with CMR-derived extra cellular volume as another myocardial tissue-based indicator for LV stiffness [99]. In this way, early diastolic impairment of myocardial function before LV deterioration or anomalies in tissue composition was demonstrated in patients suffering AMI, hypertension or amyloidosis [100–102]. RV strain impairment in patients suffering diabetes mellitus type 2 and hypertension was also shown to detect early LV diastolic dysfunction despite a preserved LVEF [103]. Of note, impaired ventricular GLS was found to correlate with myocardial fibrosis and to be an independent predictor of adverse cardiac events in patients with HFpEF [104–106]. In addition, CMR-derived T1-mapping fibrosis assessment was proven to correlate distinctly with invasively measured LV myocardial stiffness and allowed distinction of different HFpEF pathomechanisms underscoring the multiparametric possibilities of CMR imaging including tissue characterisation for the evaluation and management of these patients [107].

On the other hand, besides early diastolic deteriorations of CMR-derived ventricular strain, especially LA function, which is primarily modulated by LV contraction and has a crucial role in maintaining optimal cardiac output despite impaired LV relaxation and compliance, is an important element of detecting LV diastolic dysfunction in HFpEF [108]. Indeed, LA strain evaluations revealed especially LA reservoir strain as powerful clinical and prognostic parameters in patients with HFpEF [109]. Furthermore, decreased conduit strain is significantly associated with exercise intolerance in HFpEF patients causing impaired early ventricular filling and impaired oxygen uptake [34]. Similar results were found in RA analyses, showing RA conduit function to have strong associations with maximum oxygen uptake independent of sole RV stiffness and relaxation [110]. Moreover, RA conduit and reservoir function both were shown to be independent predictors of mortality [111]. An involvement of the right heart is often associated with more profound pulmonary vascular dysfunction and pulmonary hypertension as an expression of later stages of HF, therefore, contributes significantly to poor prognosis [112]. Indeed, CMR-FT RV and RA analyses were shown to correlate with invasively measured pressure-volume loops encouraging imaging specialists to attribute greater importance to the right heart involvement in HF patients for a more comprehensive understanding of right heart function and stiffness properties as well as a maladaptation to post-capillary pulmonary hypertension in HF [113]. Detecting functional impairments at early stages of HFpEF, recently, a study identified especially LA failure during exercise stress as a key feature in these patients [114]. LA LAS emerged as the strongest predictor for the diagnosis of diastolic HF in this work and might even serve as a future important non-invasive alternative diagnostic method in this patient collective outperforming right heart catheterization and echocardiography.

## Congenital Heart Defects

Due to special anatomical conditions, congenital heart defects are particularly challenging for cardiovascular imaging and deformation quantification. Frequently, unique morphology and structures or even converse relations of cardiac chambers, valves or vessels are necessitating deformation analyses beyond LV performance quantification in these patient collectives. Consequently, reference values and applications of CMR-FT have been already extended to pediatric and congenital diseases [24, 115, 116].

In the most common cyanotic congenital heart disease tetralogy of Fallot (ToF), despite a preserved RVEF, systolic and diastolic dysfunction were measured by CMR-FT strain and enabled diagnosis and monitoring of subclinical RV contractility and relaxation abnormalities [117]. Furthermore, impaired SR values were demonstrated to predict the occurrence of cardiac arrhythmia in these patients [118]. Comparing both CMR and echocardiography-derived biventricular FT analyses in ToF patients, a considerable intermodality variability has been demonstrated. Although echocardiographic images were shown to have higher resolution and frame rates than those of CMR, the latter enabled more reliable FT analyses due to the greater signal-to-noise ratio suggesting advantages of CMR imaging over echocardiography for these purposes [119].

As the second common acyanotic congenital heart defect, an atrial septal defect (ASD) can cause right heart dilatation but nevertheless is often tolerated well. However, depending on the size of the defect, left-right shunts in these patients can also cause cardiac remodeling and RV overfilling that result in systolic dysfunction of the RV and was shown to be measurable applying CMR-FT analyses [120]. In patients with hypoplastic left heart syndrome, CMR-FT-based RV strain assessments showed impaired deformation in those with globular/miniaturised LV morphology compared to patients with absent/slit-like LV morphology, representing a dyssynchronous septal deformation and encouraging closer monitoring and lower thresholds to start HF medications in these collectives [121]. Since LVEF is less suitable in functionally univentricular hearts, CMR-FT deformation analyses of both the dominant and the hypoplastic ventricle revealed significantly worse strain values than measurements of the dominant ventricle alone representing more accurate assessments of the total ventricular performance [122]. Moreover, despite a preserved LVEF impaired CMR-FT-based strain parameters in patients with repaired coarctation of the aorta identified adverse long-term consequences of surgical or transcatheter interventional therapy attributing CMR-FT deformation indices an important role for the clinical follow-up of these patients [123].

With the ability for precise depiction of complexly altered anatomy and enabling challenging performance quantification of distinctly changed cardiac deformation, CMR imaging and FT-based analyses are indispensable tools for congenital heart defects and benefits of this imaging technique are particularly evident in these diseases.

## Future Perspectives and Applications of CMR-Based Deformation Analyses

Numerous refinements have already turned CMR imaging into an established and indispensable imaging method over the last years. However, new developments and efforts move conventional CMR deformation assessments towards 3-dimensional (3D) analyses and implement neuronal networks for an automated and facilitated post-processing routine [124–126].

Although CMR-FT is traditionally performed on 2-dimensional (2D) images, new algorithms permit 3D CMR-FT of SSFP images and reference ranges have already been set [127]. Several advantages of this approach are obvious: on a 3D level, tracked features can be simultaneously traced in all directions and are more independent of characteristics of a single image plane. In this way, through or out of plane displacements of myocardial structures during end-systole or -diastole are less problematic [128] and deformation analyses reflect a more precise assessment of combined myocardial motions. Moreover, evaluation of complex myocardial structures and challenging tracking such as RV or atrial deformation could be facilitated [129]. To date, 3D CMR-FT analyses are principally based on two different methods. On the one hand, conventional 2D SSFP images can be used to calculate a 3D model by interpolating myocardial borders and amalgamating 2D-based deformation parameters. Thus, either 3D-based global strain values, that are comparable with common 2D parameters, can be generated [130] or new 3D principle strains depicting main myocardial motion independently from traditional directions can be estimated [124]. First applications in patients suffering HF thereby showed superior diagnostic performance compared to 2D analyses [131]. On the other hand, “true” 3D SSFP images could be acquired for calculations of an unrestrictedly orientated 3D model and subsequent deformation analysis. However, currently, CMR-FT analyses based on this technique are still limited due to temporal and spatial resolutions and its feasibility especially for challenging assessments of RV and atrial compartment need to be addressed by future studies.

Regardless of dimensionality, artificial intelligence (AI) and its applications in cardiac imaging are on the rise. First software for automated post-processing of CMR images and subsequent deformation analyses have been introduced and utilized successfully [132, 133]. As a result, post-processing times can be reduced, higher work efficiency might lead to significant cost reduction and results are less prone to observer variability [134]. Importantly, AI techniques are able to simultaneously evaluate deformation, cardiac volumes and ejection dynamics and may provide post-processing parallel to finishing the CMR protocol [135, 136]. However, these applications have been restricted to LV analyses mainly and automated analyses especially of RV or atrial analyses have not been shown to be feasible yet but might be subject to future refinements in AI software. Furthermore, applications of machine-learning techniques might perform 3D deformation analyses and future developments of this technique could not only ensure a more efficient working routine but also automatically develop novel complex protocols including compositions of all obtainable parameters and exploit new algorithm-based indices for optimised risk stratification [137].

Therefore, a profound impact of the new directions and developments of CMR-based deformation analyses can be assumed and may revolutionize not only post-processing routine but also clinical applications and usability of this imaging tool to optimise diagnostic and prognostic implications of its imaging biomarkers in the HF continuum.

## Conclusion

The current review highlights the important role of CMR-FT-derived deformation analyses for LV quantification and beyond as useful and well-established imaging tools in HF quantification. CMR-FT is attracting increasing attention of cardiovascular imaging specialists and a variety of studies have generated substantial evidence of its diagnostic and prognostic benefits. Utilizing unique CMR properties, which combine capabilities of CMR-FT for comprehensive evaluation of biventricular and atrial mechanics with myocardial tissue characterisation using LGE, T1- and T2-mapping as well as ischemia testing, provides a comprehensive whole heart all-cardiac chamber approach. Future refinements and developments of CMR-FT including multidimensional imaging and AI-based algorithms are promising and might pave the way for an even more holistic and precise personalized myocardial performance analysis technique.
